# Awareness of breast cancer among adolescent girls in Colombo, Sri Lanka: a school based study

**DOI:** 10.1186/1471-2458-13-1209

**Published:** 2013-12-20

**Authors:** Hasanthika M Ranasinghe, Nilakshika Ranasinghe, Chaturaka Rodrigo, Rohini De A Seneviratne, Senaka Rajapakse

**Affiliations:** 1 Department of Clinical Medicine, Faculty of Medicine, University of Colombo, 25 Kynsey Road, Colombo 08, Sri Lanka; 2 Department of Community Medicine, Faculty of Medicine, Colombo, Sri Lanka

**Keywords:** Adolescents, Breast cancer, Breast self examination

## Abstract

**Background:**

Breast cancer is the commonest cancer in women worldwide. Although programmes promoting breast cancer awareness are being carried out throughout Sri Lanka, few have targeted school students. We conducted this study to assess the knowledge, attitudes and practices regarding breast cancer with reference to screening, services available, breast self-examination, and sources of information, among adolescent schoolgirls in the Colombo District of Sri Lanka.

**Methods:**

The knowledge, attitudes and practices related to breast cancer were assessed among 859 adolescent girls in schools within the Colombo District, using a self-administered questionnaire. Classes and students were selected using multi-stage stratified cluster sampling.

**Results:**

Of the total sample, approximately 60% of respondents identified ‘history of breast lump’, ‘family history of breast cancer’ & ‘exposure to irradiation’ as risk factors for breast cancer. Although most were aware that the presence of a breast lump was an important warning sign, awareness of other warning signs was poor. Only 35.6% identified mammogram as an effective screening method. One third of the sample maintained that they are unaware of symptoms, diagnostics and treatment of breast cancer. Of those who were aware, 90.6% named surgery as a treatment option for breast cancer, 79.4% were unaware that chemotherapy is used. Of the total sample, 17.1% knew how to perform breast self-examination, and only 9.4% were aware of currently available breast cancer screening services. Knowledge was significantly better among students who had a relative with breast cancer.

**Conclusions:**

There were significant deficiencies in knowledge, attitudes and practices on breast cancer in the study population. In particular, knowledge on breast self examination was poor. There is a need for awareness programs aimed specifically at this important target group.

## Background

Breast cancer is recognized as the commonest cancer in females, and the second commonest malignant tumour, after lung cancer, in overall figures worldwide [1]. The incidence of breast cancer in Sri Lanka is 7.7 per 100,000 [2]. Approximately 1500 cases of breast cancer are diagnosed annually, and many are diagnosed at late stages, due to lack of awareness and the lack of a formal screening program.

The main risk factor for breast cancer is ageing. Other risk factors include a previous history of breast cancer, the use of hormone replacement therapy, high cholesterol diet and obesity [3,4]. Mammography is considered the most appropriate screening test to detect early stages of breast cancer [5].

Knowledge regarding breast cancer has been shown to be better among the more educated, and is generally satisfactory in developed countries [6,7]. Awareness and health seeking practices have been shown to be poor in many developing countries, necessitating the need for proper awareness programs [8,9]. The only published study from Sri Lanka, conducted in a population of Tamil immigrants, identified poor knowledge and cultural conservatism as barriers in implementing breast cancer screening programmes [10].

Breast cancer accounts for 45% of all cancer in females aged 25–49 years and 34% of all cancer in the 50–74 year age group in the United Kingdom [11]. The incidence in the age group between 15–24 is 3.1 per million of population in the UK [12]. In the United States, the probability of developing breast cancer remains at 0.5% for women aged less than 39 years and 3.8% for women aged 40–59 years [3]. Statistics for young women in Sri Lanka are not available. Though breast cancer is rare in younger age groups, it is generally more aggressive in this category, with lower survival rates [13]. An international survey showed poor awareness of risk factors for breast cancer among university students from 23 countries, compared to older women [14]. This emphasizes the importance of promoting breast cancer awareness among young women. Furthermore, educating the youth on breast cancer is a potential strategy for dissemination of such information in society.

Although breast cancer awareness programs are being carried out throughout Sri Lanka, these have not primarily targeted school students. We conducted this study to evaluate the knowledge, attitudes and practices related to breast cancer with reference to screening, services available, breast self examination (BSE), and sources of information, among adolescent schoolgirls in Sri Lanka.

## Methods

This was cross-sectional survey of adolescent schoolgirls in the Colombo District of Sri Lanka. General Certificate of Education (GCE) Advanced Level (AL) students (aged 17–19) studying science, mathematics, commerce, and arts subjects in selected girls’ schools of Colombo District were included in the study. Students with special needs, learning disorders or disabilities were excluded. We calculated a sample size of 769 based on the standard formula for cluster sampling, with a 95% confidence interval, response distribution of 50%, margin of error of 5%, and a design effect of 2. Allowing for a non-response rate of 5%, the final calculated sample size was 800.

Multi-stage stratified cluster sampling was used to select participants. Girls’ schools in Colombo were stratified according to school category, i.e., 1ab, and 1c. Category 1ab schools have AL classes in all 4 subjects. Category 1c schools have AL classes only in arts and commerce. Each class had approximately 30 students; a class of 30 was taken as a cluster. The numbers of students were 9323 and 1429 in categories 1ab and 1c respectively. The number of clusters was determined according to this ratio, giving 23 clusters in category 1ab schools, and 4 clusters in category 1c schools. Within each category, clusters (i.e., classes) were selected using random sampling, proportionate to the subject streams (i.e., category 1ab: arts 5, commerce 6, mathematics 6, science 6; category 1c: arts 2, commerce 2; total: 23). All students in the selected clusters present in school on the day of the survey were enrolled.

The data collection instrument was a self-administered questionnaire developed by the authors; it was modified based on findings of focus group discussions. The questionnaire was made available in all 3 national languages, i.e., Sinhala, Tamil & English, and consisted of 11 sections, with a total of 60 items. Questions on demography, knowledge regarding causes and risk factors of breast cancer, knowledge of early warning signs of breast cancer, knowledge of early detection measures including breast self examination, and sources of information, were included. Data was analyzed using SPSS^®^ 18.0 software. Knowledge was scored using percentages, and univariate analysis was used for comparisons.

Ethics approval was obtained from the Ethics Review Committee of the Faculty of Medicine, University of Colombo. Written permission was obtained from the Director of Education and Zonal/Provincial Directors of Education. Permission was also obtained from the school principals and class teachers. Informed verbal consent was taken from the participants prior to enrollment. Those who were not willing participate were not given the questionnaires. The data collection was anonymous.

## Results

The survey enrolled 859 female students from seven girls’ schools in the Colombo district. Of the total sample, 466 (54.2%) were Grade 12 students and 393 (45.8%) were Grade 13 students. Ethnically, 93% were Sinhalese, 3.7% were Tamils and 2.2% were Muslims. Most of the participants were residents of the Colombo district (79.5%) while 13.3% were from the Gampaha District and 4.4% were from the Kalutara district (the Gampaha and Kalutara districts outlie the Colombo district). Forty four (5.2%) students had a family history of breast cancer.

### Early warning signs of breast cancer

Student responses on early warning signs are summarized in Table 1.

**Table 1:** Knowledge on early warning signs of breast cancer

	**Correct answer (Percentage)**	**Wrong answer (Percentage)**	**Don’t know (Percentage)**
1) Breast lump	72.4	10.7	16.9
2) Pain in one breast	52.3	19.4	28.3
3) Cyclical/monthly pain in both breasts	35.1	16.5	48.4
4) Recent onset nipple discharge in a non-pregnant woman	35.8	16.5	47.7
5) Recent onset nipple retraction/inversion	21.3	22.7	56.0
6) Lump in neck or armpit	19.0	45.6	35.4
7) Skin changes of breast	47.2	21.9	30.9
8) Asymmetry of the breasts since childhood	57.3	10.1	32.6

When asked what their first action would be if they ever felt a breast lump in themselves, the majority of students (57.8%) stated that they would inform a family member, while 23.6% stated that they would consult a doctor (Table 2).

**Table 2:** Response to finding a breast lump

		**Frequency**	**Percent**	**Valid percent**
Valid	Wait and see	6	.7	.7
	Inform a family member	491	57.2	57.8
	Consult a doctor if features positive for breast CA	56	6.5	6.6
	Definitely consult a doctor	203	23.6	23.9
	Seek Ayurvedic treatment	0	0	0
	More than 1 responses given	94	10.9	11.1
	Total	850	99.0	100.0
Missing	(Not answered)	9	1.0	
Total		859	100.0	

### Risk factors for breast cancer

Students’ knowledge regarding risk factors for breast cancer is shown in Table 3. Over two-thirds of students’ were aware of the relationship between breast cancer and established risk factors. Nonetheless, a large proportion appeared to be unaware of some important risk factors such as hormone replacement therapy.

**Table 3:** Knowledge on risk factors for breast cancer

**Risk factor**	**Correct answer (Percentage)**	**Wrong answer (Percentage)**	**Don’t know (Percentage)**
Older age (>45 years) is a risk factors for breast cancer	27.5	46.3	26.2
Early menarche (<12 years)	5.7	63.8	30.5
Late menopause (>55 years)	12.2	38.9	48.9
Having no children	18.9	45.5	35.6
Having more than 5 children	50.6	7.5	41.9
Late age at first child birth	16.8	35.5	47.7
Oral contraceptive use	34.8	15.1	50.1
Breast feeding	68.8	9.0	22.2
Smoking	46.9	22.4	30.7
Alcohol	40.4	25.0	34.6
Diabetes	16.4	36.8	46.8
Past history of breast lumps	67.9	5.9	26.2
Family history of breast cancer	67.5	14.8	17.7
Irradiation	74.7	5.0	20.3

### Knowledge of early detection measures for breast cancer

Knowledge of methods for early detection of breast cancer was poor, with just 81 students (9.4%) being aware of any early detection method. Forty three students (5.5%) correctly identified the places where help could be sought for early detection of breast cancer, naming government and private hospitals, the National Cancer Institute Breast Care Centre, and the local public health midwife.

### Practice of early detection methods

Only 146 (17.1%) students were aware of how to perform BSE, and only 53 (6.17%) of them had ever performed one. The reasons for not performing BSE are shown in Table 4. Nearly half the students stated that they never felt the need to do BSE. Students who had a family history of breast cancer were significantly more likely to be aware of BSE compared to those without a family history (Pearson Chi-square, p = 0.033). Students studying in the science stream had a better knowledge of BSE. Students in two schools (Visakha Vidyalaya and Samudradevi Balika Vidyalaya) had better knowledge on BSE, because they had had a recent seminar on the topic. Regarding how frequently BSE should be done, 37% answered ‘once a month’, and 28.3% answered ‘once in 3 months’, 10.8% stated ‘once a year’ and 12.8% stated that they did not know.

**Table 4:** Reasons for not performing BSE

		**Frequency**	**Percent**	**Valid percent**
Valid	Never felt necessary	421	49.0	56.4
	Don’t know how to do	317	36.9	42.4
	I feel shy to do it	3	.3	.4
	Unaware of proper/complete technique	3	.3	.4
	Cancer risk is high only after 45 years	1	.1	.1
	I did not think young people can get cancer	1	.1	.1
	Haven't heard of it before	1	.1	.1
	Total	747	87.0	100.0
Missing	(not answered)	112	13.0	
Total		859	100.0	

### Treatment options for breast cancer

One third of the sample was unaware of treatment options for breast cancer. Of those who were aware, 90% of the respondents identified surgical intervention as a treatment option. Student response in this regard is summarized in Table 5.

**Table 5:** Knowledge on treatment of breast cancer

	**Yes (Percentage)**	**No (Percentage)**	**Don’t know (Percentage)**
1) Surgery	90.6	2	7.3
2) Radiotherapy	57.7	10.7	31.2
3) Chemotherapy	11.1	8.7	79.4
4) Injections to breast	27.6	20	52
5) Hormonal pills	11.8	33.2	54.7
6) Local applications/balms	15.8	35.9	48.2

### Source of information

**Figure 1:**
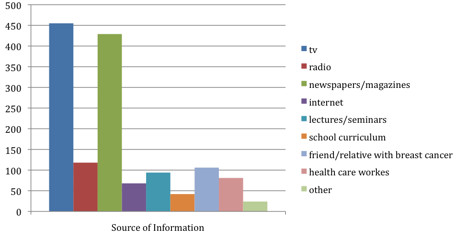
Sources of information regarding breast cancer.

Television and newspapers were cited as the most important sources of information regarding breast cancer (Figure 1).

## Discussion

Developing proper health practices should commence as early as possible, and should lead to lifetime maintenance of good health. Adolescent females are an important target group for promotion of proper health habits, in particular with regards to breast health. To our knowledge there are no published data on knowledge, attitudes and practices among adolescent females on breast cancer in Sri Lanka, and few published studies worldwide [15,16].

The knowledge regarding risk factors for breast cancer in this sample was poor. The majority of respondents were not aware of early warning signs such as nipple changes and a lump in the armpit. The knowledge on screening methods was also unsatisfactory with a majority being unaware of either mammogram or BSE.

Notably, students from two schools where recent seminars on breast cancer had been held had better knowledge, especially of BSE. This seems to be an effective mode of education. Students also made suggestions to distribute printed leaflets and to incorporate information about breast cancer into the school curriculum in the subject of health sciences in the GCE Ordinary Level (OL) classes (grades 9–11). The latter seemed to be more appropriate than including it during AL classes as some students leave school after completing OL classes. Mass media such as television and newspapers were cited as sources of information by many, but such means of communication appeared largely ineffective, judging by the degree of unawareness on core knowledge. A more individualized approach allowing two-way communication seems to be more effective.

The findings of this study shed light on the fallacy of complacency of having health education “in the books” and expecting students to be knowledgeable about it. The process of imparting knowledge effectively can be hampered by many factors such as ineffective teaching, lack of understanding by the recipient, cultural taboos and social pressure barring teaching in certain settings (such as mixed gender schools). Even if knowledge is acquired it does not necessarily transfer positively into attitudes and practice. For example, in this sample 17% claimed to know how to perform BSE, but only one third of them had ever attempted it.

Similar studies have evaluated the knowledge of women on BSE in other countries. A study of 200 women aged 20 or older in Jeddah, Saudi Arabia showed that nearly 80% had heard about BSE, but only 48% knew how to do it [17]. A larger study in Northern Saudi Arabia also showed general awareness of breast cancer and BSE to be poor [18]. A knowledge, attitudes and practices (KAP) survey in Ghana (n-232) showed that a formal educational programme can significantly improve knowledge on BSE [19]. In Karachchi, Pakistan, a survey of 373 participants showed that 49% of the sample had heard about BSE, 38% knew how to do one but only 26% had ever done it [20]. A meta-analysis of 18 studies of BSE in Turkey conducted between 2000 – 2009 showed that married women and those who have had a family member with breast cancer were more likely to perform BSE [21]. A survey of young college students in UK showed that greater perception of hindrances to BSE and also having a higher severity perception of breast cancer were characteristics of women who were not doing BSE despite being knowledgeable about it [22]. However, studies have also shown that improved literacy rates in general positively influences positive health behaviour towards breast cancer [23].

While it is accepted that the standards of teaching with regard to breast cancer should improve, there are doubts as to who are the best teachers to teach the students. Class teachers may not be the best option in this regard for two reasons; a) unfamiliarity with the subject and b) cultural sensitivity (especially teaching the topic in non-gender segregated schools) which might leave them uncomfortable to discuss the topic. There are no studies from Sri Lanka in this regard, however a study in Iran showed that school teachers were reasonably aware of breast cancer risk but the percentage actually performing BSE was very small (6% out of a sample of 578 women) [24]. The way out is to employ public health midwives and public health nurses who are the grass root level healthcare workers in the community. However they may also need proper training before undertaking this task as the experience in some countries is that level of knowledge on breast cancer and BSE is rather unsatisfactory among healthcare workers as well [25].

One limitation of this study is that it was confined to one district of the country. However, this is the most populous district of Sri Lanka (where the capital city Colombo is located) and students in the Colombo district have better access to information and health services in schools than in other districts. Therefore it is reasonable to assume that the results would be similar if not poorer in other areas of the country, though this fact has to be confirmed by further studies.

At the end of the study we provided participants with an educational brochure on breast cancer, and held a series of discussions with them to answer queries on the subject. Overall, our findings urge the healthcare providers and educationists to rethink their strategy of imparting knowledge to adolescents on early diagnosis of breast cancer. It is a timely need given the rising statistics of breast cancer among females in Sri Lanka, which at least partly can be prevented by screening which begins at home with BSE. It may be argued that since the expected prevalence of breast cancer in adolescent girls is low and therefore BSE has little relevance in that age group. However, targeting adolescents in health education has far reaching implications in a country like Sri Lanka. As these girls grow older, breast cancer becomes the most common cancer in females; health education in schools is a key strategy that the government can use ensure that all females in the country are educated in this regard, school education is free and compulsory for all children in Sri Lanka. The government has no other opportunity to educate all females in the country in any other stage in their life after school. Teaching BSE effectively via mass media is a challenge due to cultural reasons. Adolescent schoolgirls can also play an important role in promoting methods for early detection of breast cancer, and in particular BSE, as they would disseminate their knowledge to older female family members and friends. If more and more are educated a critical mass of knowledgeable females will exist in the community that will enable a sustainable knowledge transmission.

It is also possible that vigorously promoting BSE among adolescents can raise some concerns. For example, while adolescents are less likely than elderly women to have an underlying breast malignancy, they may have benign breast lumps such as fibroadenomata, the detection of which can cause considerable anxiety and unnecessary investigations and surgery, at great cost to the patient and to the healthcare system. At the same time BSE is not the best method of screening, since by the time a malignant growth becomes palpable it may have already spread via blood and lymphatics. However, in a resource limited setting like ours, an alternative screening method (e.g. mammogram) is too costly to be standard practice and is also technically inappropriate in adolescents. Considering all this we argue that BSE is a key skill that needs to be taught to adolescents because it is a quick and simple procedure that costs nothing to the patient and because, having learned the skill early in life, the girls can through their adult life practicing it and teaching it to others.

## Conclusion

This study provides important baseline information regarding the knowledge on breast cancer, screening services and breast self examination among adolescent schoolgirls in Colombo, Sri Lanka. Overall, the knowledge of these among our target population is poor. We recommend that health professionals should target this important group, by introducing effective breast health programmes to help adolescent females develop good health habits early on.

## Abbreviation

BSE: Breast self examination.

## Competing interests

The authors declare that they no competing interest.

## Authors’ contributions

SR, HMR and NR proposed the idea for the study and developed the initial protocol. RDAS developed the study design and the sampling protocol. HMR and NR collected and entered data, and did the initial analysis. SR and HMR finalized the statistical analysis and wrote the first draft. CR reviewed and refined the manuscript. All authors read and authorized the final manuscript.

## Authors’ information

HMR (MBBS) and NR (MBBS) were research associates, CR (MD) is lecturer, and SR (MD) is Professor in the Department of Clinical Medicine, Faculty of Medicine, University of Colombo, Sri Lanka. RDAS (MD) is Professor in the Department of Community Medicine, Faculty of Medicine, University of Colombo, Sri Lanka.

## Pre-publication history

The pre-publication history for this paper can be accessed here:

http://www.biomedcentral.com/1471-2458/13/1209/prepub
